# ILC2s mediate systemic innate protection by priming mucus production at distal mucosal sites

**DOI:** 10.1084/jem.20180610

**Published:** 2019-10-03

**Authors:** Laura Campbell, Matthew R. Hepworth, Jayde Whittingham-Dowd, Seona Thompson, Allison J. Bancroft, Kelly S. Hayes, Tovah N. Shaw, Burton F. Dickey, Anne-Laure Flamar, David Artis, David A. Schwartz, Christopher M. Evans, Ian S. Roberts, David J. Thornton, Richard K. Grencis

**Affiliations:** 1 Wellcome Trust Centre for Cell Matrix Research, Faculty of Biology, Medicine and Health, University of Manchester, Manchester Academic Health Science Centre, Manchester, UK; 2 Lydia Becker Institute of Immunology and Inflammation, Faculty of Biology, Medicine and Health, University of Manchester, Manchester Academic Health Science Centre, Manchester, UK; 3 Manchester Centre for Collaborative Inflammation Research, School of Biological Sciences, Faculty of Biology, Medicine and Health, University of Manchester, Manchester Academic Health Science Centre, Manchester, UK; 4 Department of Pulmonary Medicine, The University of Texas MD Anderson Cancer Center, Houston, TX; 5 Jill Roberts Institute for Research in Inflammatory Bowel Disease, Joan and Sanford I. Weill Department of Medicine, Department of Microbiology and Immunology, Weill Cornell Medicine, Cornell University, New York, NY; 6 University of Colorado, School of Medicine, Department of Medicine, Aurora, CO; 7 University of Colorado Denver School of Medicine, Division of Pulmonary Sciences and Critical Care Medicine, Aurora, CO

## Abstract

Host immunity to parasitic nematodes requires the generation of a robust type 2 cytokine response, characterized by the production of interleukin 13 (IL-13), which drives expulsion. Here, we show that infection with helminths in the intestine also induces an ILC2-driven, IL-13–dependent goblet cell hyperplasia and increased production of mucins (Muc5b and Muc5ac) at distal sites, including the lungs and other mucosal barrier sites. Critically, we show that type 2 priming of lung tissue through increased mucin production inhibits the progression of a subsequent lung migratory helminth infection and limits its transit through the airways. These data show that infection by gastrointestinal-dwelling helminths induces a systemic innate mucin response that primes peripheral barrier sites for protection against subsequent secondary helminth infections. These data suggest that innate-driven priming of mucus barriers may have evolved to protect from subsequent infections with multiple helminth species, which occur naturally in endemic areas.

## Abstract (precis)

Campbell et al. show that intestinal helminth infection generates mucin-mediated host protection at distal mucosal sites driven by interleukin-13 from innate lymphoid cells. This provides an important innate defense mechanism operating against the multiple helminth challenges encountered at mucosal surfaces.

## Introduction

While it is widely accepted that adaptive immunity is key to control of multicellular pathogens across barrier surfaces (Grencis, 2015), it is becoming increasingly clear that innate responses resulting from damage caused by pathogen invasion can play a critical role, particularly at mucosal sites such as the lung and intestinal tract (Spits et al., 2013; Walker et al., 2013). Type 2 immune responses are critical in driving expulsion of helminths via induction of goblet cell hyperplasia and mucus secretion (Oeser et al., 2015). The primary protective surface at mucosal sites is the secreted mucus barrier, which is a dynamic multimolecular matrix built on polymeric, gel-forming glycoproteins (mucins), with different mucins dominating the barrier at different mucosal sites (Thornton et al., 2008). At mucosal sites, specialized epithelial cells such as goblet cells secrete gel-forming mucins. Upon infection, these cells undergo hyperplasia and increase mucin production, which expands the secreted mucus barrier and provides protection against multiple pathogens (Else and Finkelman, 1998; Khan et al., 2001; Webb et al., 2007). The mucus layer also contributes to the tissue immune response by incorporating antimicrobial substances (e.g., defensins, lysozyme, and IgA), immunomodulatory molecules (e.g., secretoglobins and cytokines), and repair molecules (e.g., trefoil proteins; Vreugdenhil et al., 2000; Thim et al., 2002; Meyer-Hoffert et al., 2008; Vaishnava et al., 2011; Wells et al., 2017).

In the intestine, the mucin Muc2 (in mice)/MUC2 (in humans) is the major gel-forming mucin, which provides a protective barrier against microbes as well as modulating antigen sampling and tolerance (Johansson et al., 2013). In the respiratory tract, the mucus layer provides hydration and protection against inhaled pathogens and toxicants. Muc5b/MUC5B and Muc5ac/MUC5AC are the major gel-forming mucins of airway mucus (Young et al., 2007; Fahy and Dickey, 2010) and contribute to the protective properties of this mucosal barrier. In mice, Muc5b is required for maintaining immune homeostasis in the lungs (Roy et al., 2014), whereas Muc5ac is up-regulated in allergic inflammation (Evans et al., 2015) suggesting the two mucins may have differing roles. Interestingly, Muc5ac is also up-regulated in the intestine following helminth infection and required for expulsion (Hasnain et al., 2011), suggesting a key role for coordinated mucus responses in immunity to helminth infections at multiple barrier surfaces.

Intestinal-dwelling helminths are ubiquitous parasites of man and animals and have played an important part in the evolution of our immune system (Maizels and McSorley, 2016). Their life cycle strategies are varied depending on species, but all involve invasion of at least one mucosal site, and often two (Zaph et al., 2014). Resistance to these infections is dependent on the generation of a robust type 2 cytokine response, in particular production of IL-13 (Grencis et al., 1991; Urban et al., 1998; Finkelman et al., 1999; Cliffe and Grencis, 2004). In addition, while a key role for CD4^+^ T cells in protection against many species of helminths is well established (Harris and Loke, 2017), more recently a major role for innate cell types in resistance has also been demonstrated, particularly for IL-13–secreting group 2 innate lymphoid cells (ILC2; Moro et al., 2010; Neill et al., 2010; Price et al., 2010; Klose and Artis, 2016). The effector mechanisms responsible for host protection against intestinal nematodes controlled by IL-13 are dominated by the effect this cytokine has on the regulation of host epithelium (Cliffe et al., 2005), most notably mucin-producing goblet cells. Indeed, we have previously shown that defined mucins (Muc2 and Muc5ac) are important in the intestinal protective response to multiple helminth species (Hasnain et al., 2010, 2011).

The conservation of type 2 mediated effector responses in immunological protection against intestinal nematodes is remarkable bearing in mind the differences between species phylogenetically. Here we show that infection with a gastrointestinal (GI) helminth also induces a systemic innate IL-13–driven mucin-mediated protective immunity, which primes distal barrier tissue sites for subsequent secondary infections with multiple different helminth species. Specifically, infection within the intestinal tract elicited ILC2s, which migrated and induced goblet cell hyperplasia and production of mucins distally in the respiratory tract. This systemic innate response in turn primed this tissue for protection against a secondary infection with a disparate species in the lungs. Importantly, we show that the elevated mucus production is sufficient to protect against helminth infection in the lung. Together, we demonstrate that infection by GI-dwelling nematodes induces innate-driven changes in the secreted mucus barrier at multiple mucosal sites regardless of the species of infecting helminth. This provides an important first-line innate defense mechanism that operates against the multiple helminth challenges that both man and animals will encounter at host mucosal surfaces.

## Results and discussion

### Intestinal nematode infection generates a rapid IL-13–driven goblet cell hyperplasia at distal, uninfected mucosal barrier sites

**Figure 1.**
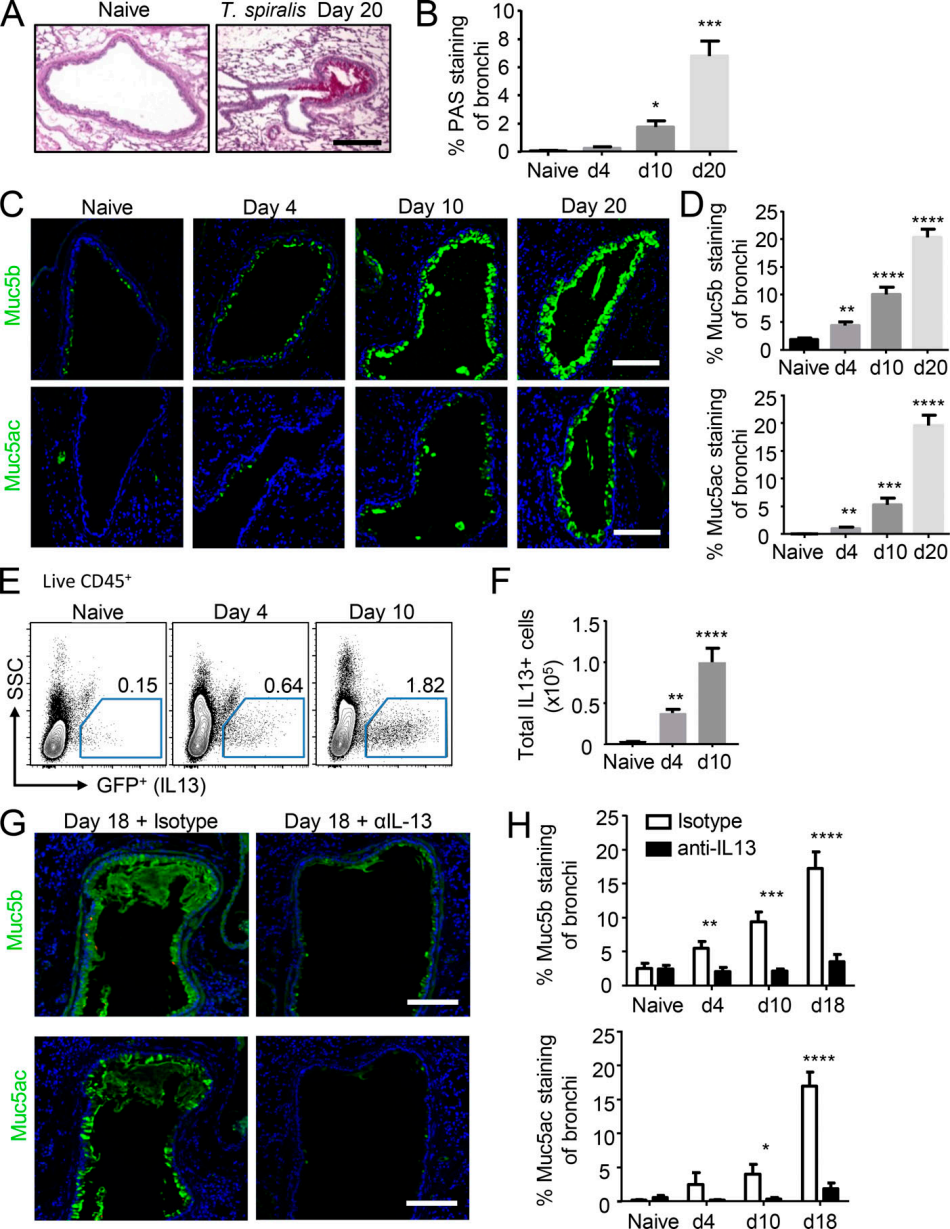
**Intestinal *T. spiralis* infection causes IL-13–mediated goblet cell hyperplasia distally in the lung. (A)** Representative PAS staining of lung tissue sections from *T. spiralis*–infected mice (bar, 100 µm). **(B)** Quantification of PAS staining over time course of infection. PAS-stained representative sections of five mice/group were analyzed. **(C)** Representative lung sections from *T. spiralis*–infected mice stained for either Muc5b (top panel) or Muc5ac (bottom panel; bars, 100 µm). **(D)** Quantification of Muc5b- and Muc5ac-stained lung sections (*n* = 5 mice/group). **(E)** Flow cytometry analysis of lung IL-13^eGFP+^ cells from infected mice. Representative dot plots are shown (pregate: singlet, live, CD45^+^ cells). SSC, side scatter. **(F)** Quantification of total lung IL-13^eGFP+^ cells (*n* = 6 mice/group). Naive and *T. spiralis*–infected mice were injected i.p. with either anti–IL-13 or isotype control (from day −1 and every 2 d until collection; 200 µg/mouse). **(G)** Representative day 18 p.i. lung sections from anti–IL-13 and isotype-treated mice stained for either Muc5b (top panel) or Muc5ac (bottom panel; bars, 100 µm). **(H)** Quantification of Muc5b- and Muc5ac-stained lung sections (*n* = 3 mice/group). Data are representative of two (H) or three (B, D, and F) independent experiments. Error bars indicate the mean ± SEM. Comparisons to either naive group (B, D, and F) or isotype control (H) were calculated using unpaired Student’s *t* tests. *, P ≤ 0.05; **, P ≤ 0.01; ***, P ≤ 0.001; ****, P ≤ 0.0001.

To investigate the effect of systemic mucosal responses to infection, we used the intestinal dwelling nematode *Trichinella spiralis*. Previous work has shown that *T. spiralis* infection results in an IL-13–driven intestinal goblet cell hyperplasia, with elevated levels of the mucins Muc2 and Muc5ac, which aids in the expulsion of the parasite from the GI tract (McDermott et al., 2005; Hasnain et al., 2011). Surprisingly, we found that mice infected with *T. spiralis* also exhibited mucus production in the lung, despite the absence of infection at this site (Fig. 1, A–D). Both Muc5b and Muc5ac protein levels were significantly increased by day 4 postinfection (p.i.), which is before migration of L1 stage *T. spiralis* larvae out of the intestinal tract to skeletal muscle (Harley and Gallicchio, 1971; Wang and Bell, 1986), and reached a peak by day 20 p.i., when the intestinal infection had long been cleared (Fig. 1, C and D; and Fig. S1 A). Moreover, at day 42 p.i., significantly increased levels of both mucins persisted within the lung, suggesting that intestinal-elicited immune responses may alter tissue function at distal uninvolved sites (Fig. S1 B). Interestingly, *T. spiralis* infection also induced changes in mucin production in the eye, a site also associated with infection/migration of various nematode species (Ahn et al., 2014), characterized by an increase in corneal Muc5b expression from day 4 p.i. (Fig. S1 C). As *T. spiralis* migrates through the blood/lymph to muscle tissues, we tested the ability of other intestinal-restricted helminth infections in priming distal mucosal sites. Importantly, the mucin response was not limited to *T. spiralis* infection and was observed in strictly intestinal-dwelling helminths (Cunningham and Olson, 2010; Maizels et al., 2012), as infection with either the mouse roundworm *Heligmosomoides polygyrus* or the intestinal dwelling cestode *Hymenolepis microstoma* also caused significantly elevated Muc5b and Muc5ac production distally in the lung (Fig. S1, D and E).

*T. spiralis*–infected mice also exhibited a significant elevation in IL-13^+^ CD45^+^ cells in the lung parenchyma (Fig. 1, E and F) as early as day 4 p.i. In vivo administration of a neutralizing anti–IL-13 antibody during infection significantly reduced both the Muc5b and Muc5ac protein levels in the lung to baseline (Fig. 1, G and H). Additionally, mice deficient for IL-13 (*Il13*^−/−^) clearly exhibited an inability to up-regulate both mucins within the lung following intestinal *T. spiralis* infection (Fig. S2 A), this establishes that IL-13 has a critical role to play in mucin up-regulation during nematode infections at distal sites, in line with previous findings implicating IL-13 in Muc5b and Muc5ac production in the mouse lung (Kuperman et al., 2002; Portal et al., 2017). Collectively, these data show that intestinal infection with helminths induces an IL-13–driven production of mucins within the airways, a site distal from infection.

### ILC2-derived IL-13 is required for lung mucin production following intestinal *T. spiralis* infection

**Figure 2.**
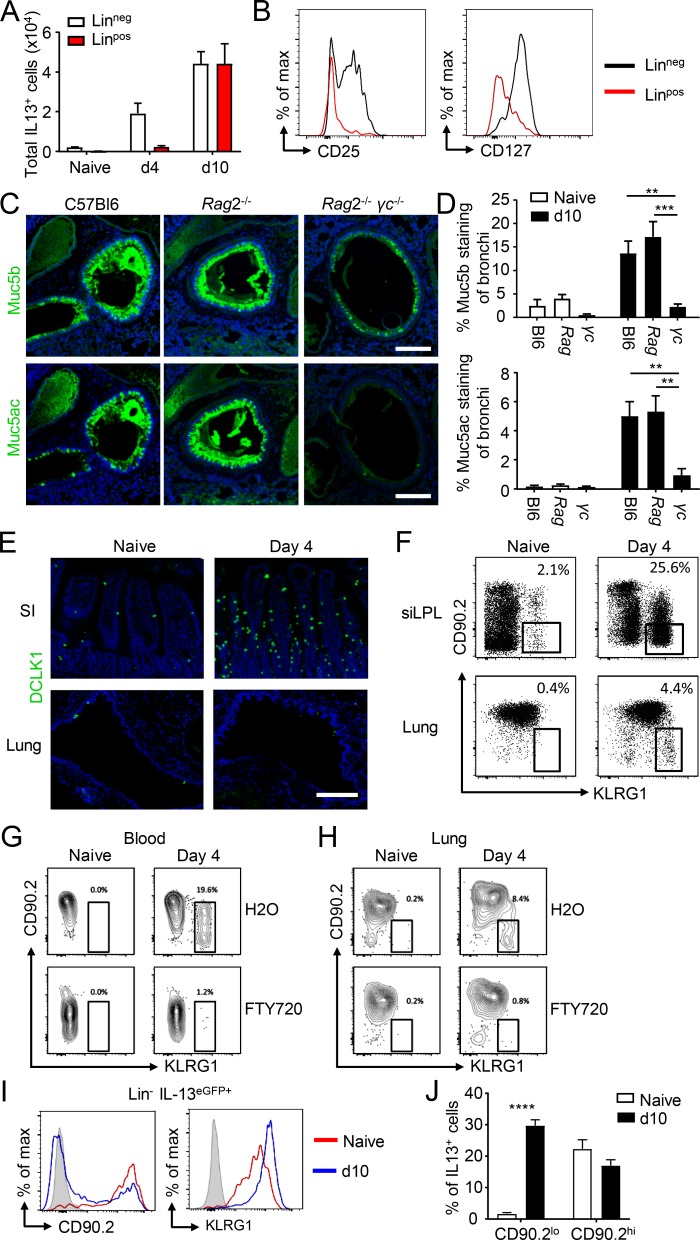
**Inflammatory ILC2-derived IL-13 is sufficient to drive lung goblet cell hyperplasia. (A)** Analysis of lung-derived IL-13^eGFP+^ cells from naive and *T. spiralis*–infected mice. Cell subsets determined by lineage expression (LIN^neg^: CD3^−^, TCRβ^−^, TCRγδ^−^, CD49b^−^, CD19^−^, B220^−^, CD11b^−^, CD11c^−^, TER119^−^, GR1^−^, FcεR1α^−^). **(B)** Expression of CD25 and CD127 on lung IL-13^eGFP+^ cells from day 10 *T. spiralis*–infected mice in the Lin^neg^ subset (pregate: singlet, live, CD45^+^, IL-13^eGFP+^ cells; *n* = 6 mice/group). **(C and D)** Representative lung sections from day 10–infected WT, *Rag2^−/−^*, and *Rag2^−/−^ γc^−/−^* mice (C) stained for either Muc5b (top panel) or Muc5ac (bottom panel; bars, 200 µm) and corresponding quantification of Muc5b- and Muc5ac-stained lung sections (D; *n* = 3 mice/group). **(E)** Representative small intestine (SI) and lung sections from naive and day 4–infected mice stained with DCLK1 (bars, 200 µm). **(F)** Cells from the small intestinal lamina propria (siLPL) and lung of naive and day 4–infected C57/BL6 mice were analyzed by flow cytometry for iILC2 markers (gated as Lin^−^ CD127^+^ CD90.2^lo^ KLRG1^hi^). **(G and H)** C57/BL6 mice were injected i.p. with either FTY720 or H_2_O (daily from day −1; 1 mg/kg). Cells from the blood (G) and lung (H) of naive and day 4–infected mice were analyzed by flow cytometry for iILC2 markers (gated as Lin^−^ CD127^+^ CD90.2^lo^ KLRG1^hi^). **(I)** Expression of CD90.2 and KLRG1 on Lin^neg^ IL-13^eGFP+^ cells from naive (red line) and day 10–infected (blue line) lungs (*n* = 5 mice/group). **(J)** Frequency of IL-13^eGFP+^ cells expressing either low or high levels of CD90.2 from naive and day 10–infected lungs (*n* = 5 mice/group). Data are representative of two (D, F, G, and H) or four (A, B, and J) independent experiments. Error bars indicate the mean ± SEM. Comparisons to naive group were calculated using unpaired Student’s *t* tests. **, P ≤ 0.01; ***, P ≤ 0.001; ****, P ≤ 0.0001.

T helper type 2 cells and ILC2s are both major producers of IL-13, although the kinetics of these responses differ (McKenzie et al., 1993; Klose and Artis, 2016). To determine the cellular source of IL-13 responsible for *T. spiralis*–induced mucin expression in the lungs, the phenotype and frequency of IL-13^eGFP+^ cells were determined following infection. 4 d p.i., the majority of IL-13^eGFP+^ cells were found to be negative for major lineage markers, suggestive of an innate cell source. In contrast, by day 10 p.i., IL-13 in the lung was derived equally from both lin^pos^ and lin^neg^ cell sources (Fig. 2 A). ILC2s are a major source of type 2 cytokines in the lung in the context of allergic airway inflammation or following infection (Chang et al., 2011; Monticelli et al., 2011; Barlow et al., 2012). At day 10, the lin^neg^ IL-13^eGFP+^ population expressed CD25 and CD127, in contrast to the lin^pos^ cells, indicative of an ILC phenotype (Fig. 2 B; Spits et al., 2013). As mucin responses were detected as early as day 4, we hypothesized that adaptive sources of IL-13 may be dispensable for systemic mucin responses to *T. spiralis*. Accordingly, *Rag*2^−/−^ mice demonstrated comparable increases in lung Muc5ac and Muc5b expression at day 10 p.i. In contrast, *Rag*2^−/−^ *γc*^−/−^ mice, which lack both adaptive cells and ILC, had severely impaired mucin responses following infection, with a complete lack of Muc5ac expression (Fig. 2, C and D). This suggested that ILC2-derived IL-13 is sufficient for systemic mucin responses in the lung following intestinal helminth infection.

ILC2s are increasingly appreciated to be phenotypically and functionally heterogeneous and can be differentially elicited by multiple alarmins and cytokines, including IL-33, IL-25, and thymic stromal lymphopoietin (Klose and Artis, 2016). Previous studies have demonstrated that while IL-33 elicits ILC2 with a classical phenotype (termed natural ILC; nILC2), IL-25 can elicit ILC2 with inflammatory potential (termed iILC2), characterized by their functional plasticity and ability to produce IL-17A, high expression of the activation marker KLRG1, and relatively low surface expression of CD90/Thy1 (Huang et al., 2015). Furthermore, recent findings indicate that IL-25 produced by intestinal tuft cells elicits a population of iILC2 with the potential to recirculate to peripheral tissues, including the lung (Howitt et al., 2016; von Moltke et al., 2016; Huang et al., 2018). To determine if *T. spiralis* infection similarly elicits tuft cells and intestinal iILC2, intestinal sections from infected mice at day 4 p.i. were stained with DCLK1. While increases in tuft cell numbers occurred rapidly within the intestine, no response was observed within the lung (Fig. 2 E). Further to this, we profiled the ILC2 phenotype within the small intestinal lamina propria and lung following *T. spiralis* infection. In line with the tuft cell response, a marked increase in ILC2s was observed in the intestinal tract at day 4 p.i., which predominantly exhibited low surface expression of CD90.2 and high KLRG1 (Fig. 2 F). Notably, infection also induced an increase in CD90.2^lo^ KLRG1^hi^ iILC2 (also ST2 low, CD25 low; Fig. S2 B) distally in the lung parenchyma at day 4 p.i., despite the lack of local tuft cell hyperplasia (Fig. 2, E and F). Administration of FTY720 over the period of infection blocked migration of this cell population into the blood (Fig. 2 G) and lungs (Fig. 2 H), supportive of their intestinal source and the previously reported migratory capacity of iILC2 (Huang et al., 2018). Moreover, increased mucin production was found to be intact in the lungs of day 10–infected *Il33*^−/−^ mice, and analysis of blood and lung CD90.2^lo^ KLRG1^hi^ iILC2 frequency was similar in day 4–infected *Il33*^−/−^ mice, suggesting this ILC2-activating cytokine is dispensable for systemic airway responses following intestinal *T. spiralis* infection (Fig. S2, C and D) and indicative of a predominant role for IL-25–induced iILC2 responses. Moreover, selective gating of IL-13^eGFP+^ Lin^neg^ cells in the lung at day 10 p.i. confirmed that CD90.2^lo^ KLRG1^hi^ iILC2 were the predominant source of cytokines in infected mice, whereas IL-13^eGFP+^ Lin^neg^ cells present in naive mice were largely CD90.2^hi^ KLRG1^hi^, a phenotype more closely associated with nILC2 (Fig. 2, I and J). Also, transfer of IL-13^eGFP+^ iILC2 from the lungs of *T. spiralis*–infected mice into immunodeficient mice lacking ILC efficiently restored the lung Muc5b and Muc5ac response (Fig. S2 E). The data, therefore, demonstrate that ILC2s are sufficient to drive mucin responses in the lung and suggest that an intestinally induced iILC2 acts to induce mucus production systemically through migration to peripheral tissues, such as the lung, in an IL-13–dependent manner. Moreover, this mechanism of mucin induction may be conserved across multiple intestinal helminth infections, as we also observed an elevation of intestinal tuft cell numbers following *H. microstoma* infection (Fig. S2 F), similar to that shown for *Nippostrongylus brasiliensis* (Nb) and *H. polygyrus* infections (Gerbe et al., 2016; Howitt et al., 2016; von Moltke et al., 2016).

### Intestinal helminth infection cross-protects against secondary infection with a heterologous helminth in the lung via goblet cell hyperplasia and increased mucus secretion

**Figure 3.**
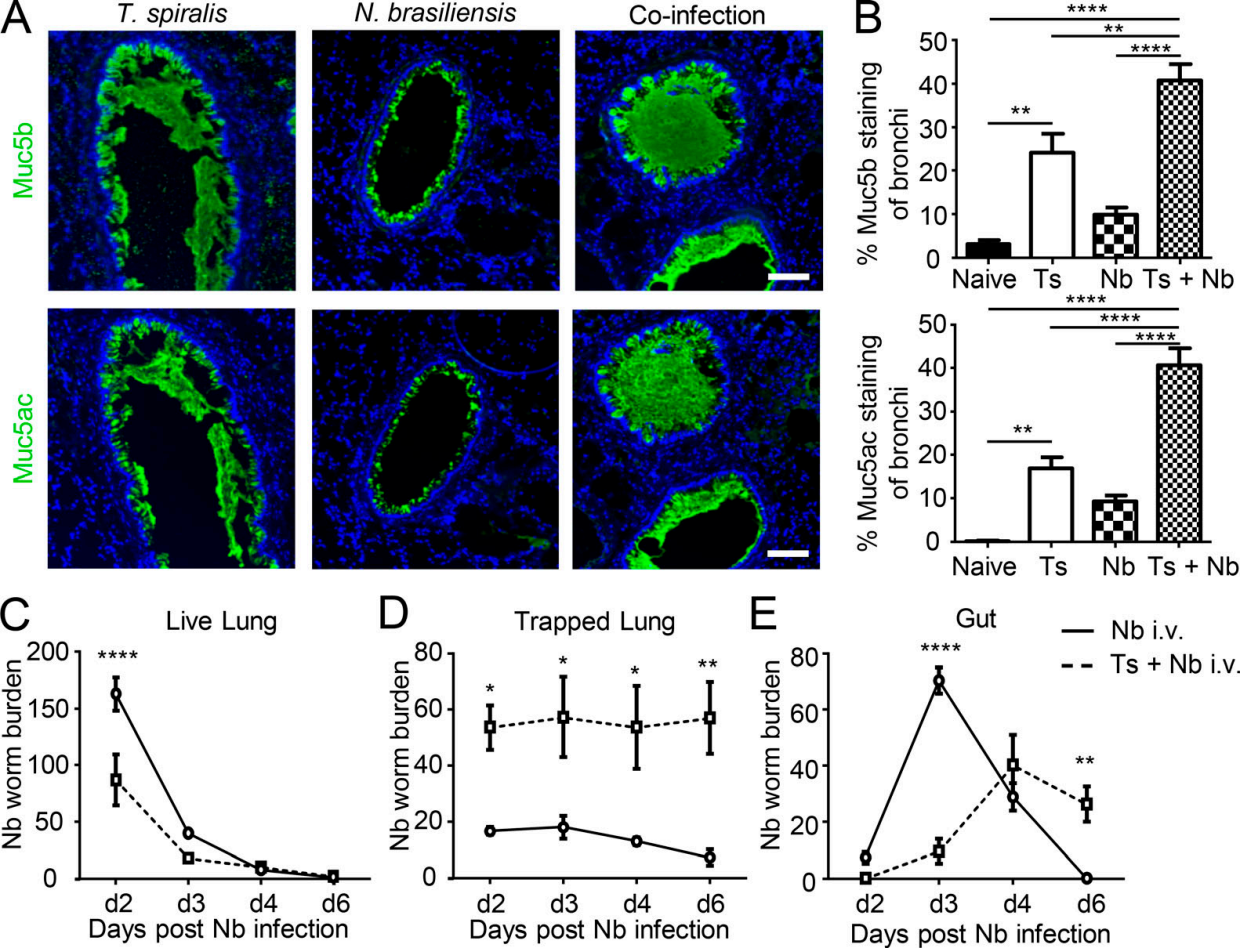
**Systemic priming by *T. spiralis* infection impedes a subsequent Nb infection in the lung. (A)** Representative lung sections from day 22 after *T. spiralis* infection, day 2 after Nb infection, and coinfected mice stained for either Muc5b (top panel) or Muc5ac (bottom panel; bars, 100 µm). **(B)** Quantification of Muc5b- and Muc5ac-stained lung sections from Nb-infected C57/BL6 mice. Naive, day 22 after *T. spiralis* (Ts), day 2 after Nb and coinfected (Ts + Nb; *n* = 3 mice/group). **(C–E)** Naive (solid line) or day 20 after *T. spiralis*–infected (dashed line) C57/BL6 mice were i.v. infected with Nb. Total Nb worm numbers were collected from lung tissue (C), collagenase-digested lung tissue (D), and small intestine (E; *n* = 3 mice/group). Data are representative of three independent experiments. Error bars indicate the mean ± SEM. Comparisons between groups were calculated using either two-way (A) or one-way (B) ANOVA and Sidak’s post-test. *, P ≤ 0.05; **, P ≤ 0.01; ****, P ≤ 0.0001.

As intestinal helminth infections induce an innate-driven mucin response distally in the lung, we postulated that this conserved host response may be protective and act to prime systemic tissues against further invading helminths. To confirm this cross-protective nature of the elevated mucus response in the lung, mice were challenged i.v. with L3 of Nb 20 d after *T. spiralis* infection. Following skin penetration and migration through the circulation, L3 Nb larvae traverse through the lung spaces as part of their life cycle to reach the trachea, where they are coughed/move up the airways and subsequently swallowed before establishment in the lumen of the small intestine, their reproductive niche (Bouchery et al., 2015). *T. spiralis* single-infected animals and coinfected (*T. spiralis* and Nb) mice showed significantly elevated Muc5b and Mc5ac production in the lung compared with naive noninfected animals or animals infected with Nb alone (Fig. 3, A and B). *T. spiralis*–primed lung goblet cell hyperplasia was associated with a decreased ability of larval Nb to migrate (Fig. 3 C), sequestration of larvae in the lung (Fig. 3 D), and reduction in total larval numbers, while intestinal parasite clearance was also delayed (Fig. 3 E). This suggests that delayed Nb migration to the intestine may be due to a decreased ability to migrate out of the lung space and trapping by airway mucins. Earlier research into the role of mucins and parasite resistance suggested a role for parasite trapping within the intestinal mucus barrier (Carlisle et al., 1991; Webb et al., 2007). Indeed, we show that at all time points following infection, there are increased numbers of larvae trapped within the lung that could only be released following digestion (Fig. 3 D).

**Figure 4.**
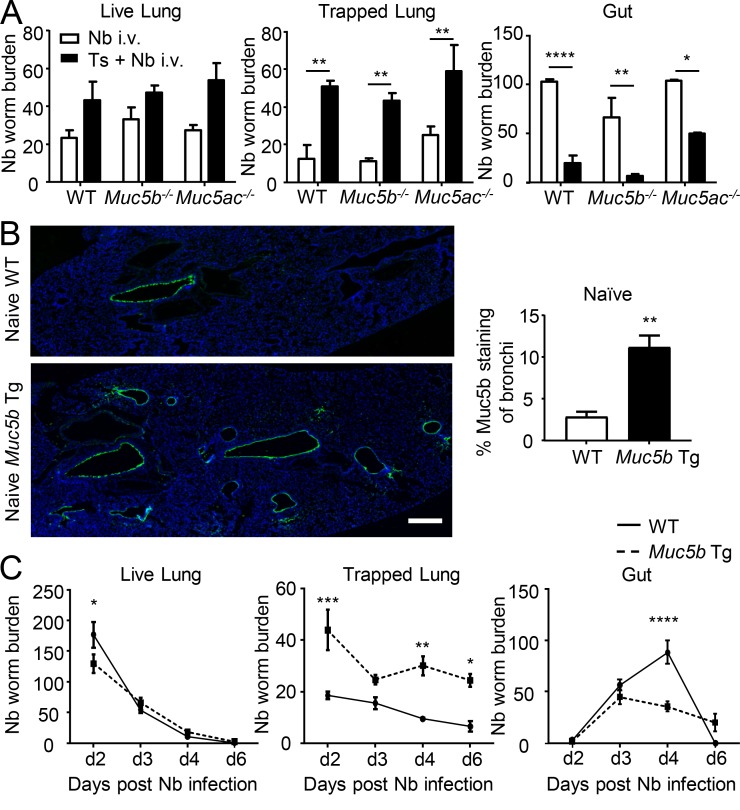
**Lung goblet cell hyperplasia protects against secondary helminth infection in the lung. (A)** Naive (white bars) or day 20 after *T. spiralis* (Ts) infection (black bars) WT, *Muc5b*^−/−^, or *Muc5ac*^−/−^ mice were i.v. infected with Nb. Total Nb worm numbers were collected at day 3 after Nb infection from lung tissue (left), collagenase-digested lung tissue (middle), and small intestine (right; *n* = 3 mice/group). **(B)** Representative lung sections from naive WT and Muc5b overexpressing (*Muc5b*-Tg) mice stained for Muc5b (bars, 400 µm) and corresponding quantification of staining (*n* = 3–4 mice/group). **(C)** WT and *Muc5b*-Tg mice were s.c. infected with Nb, and total Nb worm numbers were collected from lung tissue (left), collagenase-digested lung tissue (middle), and small intestine (right; *n* = 3–4 mice/group). Data are representative of two independent experiments. Error bars indicate the mean ± SEM. Comparisons between groups were calculated using either unpaired Student’s *t* tests (A and B) or two-way ANOVA (C) and Sidak’s post-test. *, P ≤ 0.05; **, P ≤ 0.01; ***, P ≤ 0.001; ****, P ≤ 0.0001.

To test the relative contribution of Muc5b and Muc5ac in airway larval trapping following secondary infection with Nb, similar experiments were performed in *Muc5b*^−/−^ and *Muc5ac*^−/−^ mice. Loss of either mucin was not sufficient to prevent Nb larval trapping in *T. spiralis* infection–primed mice, suggesting that Muc5b and Muc5ac may mediate redundant roles in this protective anti-helminth response (Fig. 4 A). Indeed, following *T. spiralis* infection in the specific single mucin knockout mice, we still observed markedly increased mucin production within the airways (Fig. S3 A), suggesting that sufficient mucus is produced to delay Nb transit. To test whether enhanced mucus secretion alone was sufficient to reduce Nb larval migration in the absence of prior infection, *Muc5b*-Tg mice that overexpress Muc5b were challenged with Nb L3 s.c. Naive *Muc5b*-Tg mice show significantly elevated levels of Muc5b in the lung before infection, confirming an increased mucus barrier at baseline (Fig. 4 B), in line with previous findings (Roy et al., 2014). Upon infection with Nb alone, *Muc5b*-Tg mice exhibited significantly elevated trapping of larvae, as evident from reduced passive migration out of the lung (“live lung”) and reduced migration of the parasites to the intestinal niche, as well as enhanced recovery of larvae following tissue digestion (“trapped lung”; Fig. 4 C). Thus, taken together, the data show that increased mucin production by either mucin in the lung is sufficient to significantly recapitulate the effect of prior infection, impair nematode larval migration through the airways, and limit migration and establishment of subsequent infection in the intestinal tract.

That Muc5b-overexpressing mice have the ability to limit parasite migration suggests a critical role for the mucus barrier. Previous work has indeed suggested that the intestinal mucus barrier could act to entrap parasite larvae, therefore acting as a physical barrier (Miller, 1987), as supported here by observations of mucus-trapped parasites in situ (Fig. S3 B). However, these trapping mechanisms were suggested to be antibody dependent (Carlisle et al., 1991), whereas our data would suggest this not to be the case, as mucus production was clearly evident in Rag-null mice (Fig. 2 C), as was cross-protection (Fig. S3 C), and thus immunity operates effectively in the absence of antibody and adaptive immunity. Furthermore, there is little evidence of cross-reactivity of antigens between *T. spiralis* and Nb (Kennedy, 1980). Finally, Muc5b-Tg mice were able to prevent early larval migration without prior infection, supporting the role of mucus itself as the effector. Mucus entrapment could act to inhibit parasite mobility as well as feeding capacity, rendering the parasites susceptible to clearance by other cellular components.

By investigating the systemic mucus response to different intestinal helminths, we have revealed a highly conserved innate host-protective response to invading parasites. This mechanism involves ILC2s and their effector cytokine IL-13, which have previously been shown to play important roles in the host response to infection; however, we have now shown a novel mechanism involving distal sites to the infection niche, which highlights the importance of crosstalk between multiple tissues.

## Materials and methods

### Mice

Male mice were used throughout. C57BL/6 mice aged 8–10 wk were purchased from Envigo. UK IL-13^eGFP/+^ mice on a BALB/c background and their WT (BALB/c) littermates were kindly provided by Andrew McKenzie (University of Cambridge, Cambridge, UK; Neill et al., 2010). *Il33 LacZ* gene trap reporter mice and their WT (BALB/c) littermates were kindly provided by Jean-Philippe Girard (Centre National de la Recherche Scientifique, Institut de Pharmacologie et de Biologie Structurale, Toulouse, France; Pichery et al., 2012). *Rag2*^−/−^, *Rag2*^−/−^ *γc*^−/−^, and C57BL/6 mice were purchased from Taconic. NOD/SCID*γc*^−/−^ mice were a kind gift from Dr. Santiago Zelenay (Cancer Research UK, Manchester Institute, Manchester, UK). *Muc5ac*^−/−^ and their WT (C57BL/6) littermates have been described previously (Hasnain et al., 2011). *Muc5b*^−/−^, *Muc5b ccsp*Tg^+^ overexpressers, and their WT (C57BL/6) littermates were generated as described previously (Roy et al., 2014). *Rag1*^−^*^/^*^−^ mice on a C57BL/6 background were kindly provided by John Grainger (University of Manchester, Manchester, UK). All mice were used at 8–12 wk old, and animals were euthanized using a rising concentration of CO_2_. Mice were housed in specific pathogen–free conditions, and all animal procedures were either performed under the regulations of the Home Office Scientific Procedures Act (1986) and subject to review by the University of Manchester Animal Welfare and Ethical Review Body or were approved by the Weill Cornell Medicine Institutional Animal Care and Use Committees. The experiments conform to the Animal Research: Reporting of In Vivo Experiments guidelines.

### Infections and parasite quantification

Techniques used for the maintenance, recovery, and infection of *T. spiralis* were conducted as previously described (Wakelin and Lloyd, 1976). Experimental mice were infected with 300 infectious L1 *T. spiralis* larvae by oral gavage. Techniques for the maintenance and infection of Nb were conducted as previously described (Camberis et al., 2003). Mice were infected with Nb by either i.v. or s.c. injection of 500 iL3 larvae. For coinfection experiments, coinfected mice were first infected with 300 *T. spiralis* larvae and 20 d later were infected with 500 iL3 i.v. For isolation of viable larvae from the lung, the whole lung was excised and minced with scissors, placed in gauze to form a bag, and suspended at the surface of a 50-ml polypropylene conical tube containing PBS at 37°C for 4 h. Viable worms follow a thermal gradient and migrate out of tissue to be collected at the bottom for counting. Following this, the lung tissue was digested in PBS containing 0.1 mg/ml of collagenase at 37°C for 16 h under constant agitation to assess the number of trapped/dead larvae. For isolation of adult worms, the small intestine was excised and cut longitudinally and placed in gauze to form a bag and suspended at the surface of a 50-ml tube containing PBS at 37°C for 4 h. The number of worms recovered from tissues was counted under a dissecting microscope (Leica). Methods used for the maintenance and preparation of *H. polygyrus* have been described previously (Behnke et al., 1993). Mice were infected by oral gavage with 200 infective L3 larvae. A seed culture of *H. microstoma*–infected beetles was obtained from Prof. Jerzy Behnke (Nottingham University, Nottingham, UK), and the techniques used for maintenance and infection have been described previously (Cunningham and Olson, 2010). Mice were infected with three cysticercoids by oral gavage.

### Immunohistochemistry

Freshly isolated lungs were perfused with Methacarnoy’s solution (60% absolute methanol, 30% chloroform, and 10% acetic acid), fixed in solution for 24 h, and then washed in 100% methanol before embedding in paraffin. Freshly isolated eyeballs were enucleated and fixed in Methacarnoy’s solution. 5-µm sections were stained with periodic acid–Schiff’s (PAS) reagent or subjected to immunohistochemical analysis with the following antibodies: Muc5b, generated by immunizing rabbits with synthetic peptides corresponding to the unique peptide sequence ELGQKVKCDVSSGLV, commercially available Muc5ac antibody (45M1; Sigma-Aldrich), or DCLK1 (Abcam; Ab31704). Bound primary antibody was detecting using the following antibodies; goat anti-rabbit IgG Alexa Fluor 488 (Invitrogen) and goat anti-mouse IgG Alexa Fluor 488 (Invitrogen). Sections were counterstained with DAPI. Images were captured using a Zeiss Axioimager.D2 upright microscope/Coolsnap HQ2 camera (Photometrics) through MetaVue Software. Specific bandpass filter sets for DAPI and FITC were used to prevent bleed-through from one channel to the next. Images were then processed and analyzed using ImageJ.

### In vivo treatment

Male C57BL/6 mice were infected with *T. spiralis* followed by treatment with either 0.2 mg/mouse purified murine anti–IL-13 IgG1 antibody (Genentech) or isotype control via i.p. injection. Injections were administered starting on day −1 and then every 2 d up to tissue collection. *T. spiralis*–infected male C57BL/6 mice were treated with either 1 mg/kg FTY720 (fingolimod hydrochloride; Sigma-Aldrich) or H_2_O daily throughout infection. Sort-purified IL-13^eGFP+^ ILC2 from lungs of *T. spiralis*–infected mice were divided into either CD90.2^hi^ nILC2 or CD90.2^lo^ iILC2, as per Huang et al. (2018). Sorted cytokine-positive ILC2 subsets were then transferred intranasally into naive NOD/SCIDγc^−/−^ mice.

### Cell preparation and flow cytometry

Lungs were cut into small fragments and digested for 45 min at 37°C with 1 mg/ml collagenase (Sigma-Aldrich) with gentle agitation. Red blood cells were lysed with ammonium-chloride-potassium lysing buffer. Digests were filtered twice through 70-µm cell strainers and centrifuged at 450 *g* for 5 min. Single-cell suspensions were stained with Fixable Viability Dye (eBioscience) for 30 min on ice. Fc receptors were blocked with anti-CD16/CD32 antibody (BD Biosciences) and then stained for 30 min on ice with fluorophore-conjugated antibodies. The following antibodies, purchased from BioLegend, were used to characterize IL-13^eGFP^ cells: lineage cocktail (CD3ε, TCRβ, TCRγδ, CD49b, CD19, B220, CD11b, CD11c, TER119, GR1, FcεR1α), CD45, CD25, CD90.2, CD127, ST2, and KLRG1. Flow cytometry was performed on a BD LSRII flow cytometer, and data were analyzed using FlowJo (TreeStar). Cell sorting was performed on a FACSAria.

### Statistical analysis

Prism (GraphPad Software) was used to perform all statistical analyses. Differences between groups were calculated using either unpaired Student’s *t* tests or one-way or two-way ANOVA followed by Sidak’s post hoc test for multiple comparisons (the test for each graph is specified in the figure legend). Graphs were annotated with the following markers to denote significance: *, P ≤ 0.05; **, P ≤ 0.01; ***, P ≤ 0.001; ****, P ≤ 0.0001. All graphs show mean ± SEM. All experimental data were verified in at least two independent experiments.

### Online supplemental material

Fig. S1 shows mucin analysis of day 42 lungs and day 4, 10, and 20 cornea from *T. spiralis*–infected mice, along with lung mucin analysis from *H. polygyrus*– and *H. microstoma*–infected mice. Fig. S2 shows lung mucin staining from *T. spiralis*–infected WT, IL-13^eGFP/eGFP^, and *Il33*^−/−^ mice and DCLK1 staining on *H. microstoma* small intestine sections. Fig. S3 shows lung mucin analysis in *Muc5b*^−/−^ or *Muc5ac*^−/−^ mice following infection and in situ trapping of larvae in mucus and protection in *Rag1*^−/−^ mice.

## Supplementary Material

Supplemental Materials (PDF)

## References

[bib1] Ahn S.J., Ryoo N.-K., and Woo S.J. 2014 Ocular toxocariasis: clinical features, diagnosis, treatment, and prevention. Asia Pac. Allergy. 4:134–141. 10.5415/apallergy.2014.4.3.134 25097848 PMC4116038

[bib2] Barlow J.L., Bellosi A., Hardman C.S., Drynan L.F., Wong S.H., Cruickshank J.P., and McKenzie A.N. 2012 Innate IL-13-producing nuocytes arise during allergic lung inflammation and contribute to airways hyperreactivity. J. Allergy Clin. Immunol. 129:191–8.e1: 4. 10.1016/j.jaci.2011.09.041 22079492

[bib3] Behnke J.M., Wahid F.N., Grencis R.K., Else K.J., Ben-Smith A.W., and Goyal P.K. 1993 Immunological relationships during primary infection with Heligmosomoides polygyrus (Nematospiroides dubius): downregulation of specific cytokine secretion (IL-9 and IL-10) correlates with poor mastocytosis and chronic survival of adult worms. Parasite Immunol. 15:415–421. 10.1111/j.1365-3024.1993.tb00626.x 8414644

[bib4] Bouchery T., Kyle R., Camberis M., Shepherd A., Filbey K., Smith A., Harvie M., Painter G., Johnston K., Ferguson P., 2015 ILC2s and T cells cooperate to ensure maintenance of M2 macrophages for lung immunity against hookworms. Nat. Commun. 6:6970 10.1038/ncomms7970 25912172

[bib5] Camberis M., Le Gros G., and Urban J. 2003 Animal Model of Nippostrongylus brasiliensis and Heligmosomoides polygyrus. Curr. Protoc. Immunol. Chapter 19:Unit 19.12. 10.1002/0471142735.im1912s55 18432905

[bib6] Carlisle M.S., McGregor D.D., and Appleton J.A. 1991 Intestinal mucus entrapment of Trichinella spiralis larvae induced by specific antibodies. Immunology. 74:546–551. 1769701 PMC1384653

[bib7] Chang Y.J., Kim H.Y., Albacker L.A., Baumgarth N., McKenzie A.N., Smith D.E., Dekruyff R.H., and Umetsu D.T. 2011 Innate lymphoid cells mediate influenza-induced airway hyper-reactivity independently of adaptive immunity. Nat. Immunol. 12:631–638. 10.1038/ni.2045 21623379 PMC3417123

[bib8] Cliffe L.J., and Grencis R.K. 2004 The Trichuris muris System: a Paradigm of Resistance and Susceptibility to Intestinal Nematode Infection. Adv. Parasitol. 57:255–307. 15504540 10.1016/S0065-308X(04)57004-5

[bib9] Cliffe L.J., Humphreys N.E., Lane T.E., Potten C.S., Booth C., and Grencis R.K. 2005 Accelerated intestinal epithelial cell turnover: a new mechanism of parasite expulsion. Science. 308:1463–1465. 10.1126/science.1108661 15933199

[bib10] Cunningham L.J., and Olson P.D. 2010 Description of Hymenolepis microstoma (Nottingham strain): a classical tapeworm model for research in the genomic era. Parasit. Vectors. 3:123 10.1186/1756-3305-3-123 21194465 PMC3023764

[bib11] Else K.J., and Finkelman F.D. 1998 Intestinal nematode parasites, cytokines and effector mechanisms. Int. J. Parasitol. 28:1145–1158. 10.1016/S0020-7519(98)00087-3 9762559

[bib12] Evans C.M., Raclawska D.S., Ttofali F., Liptzin D.R., Fletcher A.A., Harper D.N., McGing M.A., McElwee M.M., Williams O.W., Sanchez E., 2015 The polymeric mucin Muc5ac is required for allergic airway hyperreactivity. Nat. Commun. 6:6281 10.1038/ncomms7281 25687754 PMC4333679

[bib13] Fahy J.V., and Dickey B.F. 2010 Airway mucus function and dysfunction. N. Engl. J. Med. 363:2233–2247. 10.1056/NEJMra0910061 21121836 PMC4048736

[bib14] Finkelman F.D., Wynn T.A., Donaldson D.D., and Urban J.F. 1999 The role of IL-13 in helminth-induced inflammation and protective immunity against nematode infections. Curr. Opin. Immunol. 11:420–426. 10.1016/S0952-7915(99)80070-3 10448138

[bib15] Gerbe F., Sidot E., Smyth D.J., Ohmoto M., Matsumoto I., Dardalhon V., Cesses P., Garnier L., Pouzolles M., Brulin B., 2016 Intestinal epithelial tuft cells initiate type 2 mucosal immunity to helminth parasites. Nature. 529:226–230. 10.1038/nature16527 26762460 PMC7614903

[bib16] Grencis R.K. 2015 Immunity to helminths: resistance, regulation, and susceptibility to gastrointestinal nematodes. Annu. Rev. Immunol. 33:201–225. 10.1146/annurev-immunol-032713-120218 25533702

[bib17] Grencis R.K., Hültner L., and Else K.J. 1991 Host protective immunity to Trichinella spiralis in mice: activation of Th cell subsets and lymphokine secretion in mice expressing different response phenotypes. Immunology. 74:329–332. 1836201 PMC1384613

[bib18] Harley J.P., and Gallicchio V. 1971 Trichinella spiralis: migration of larvae in the rat. Exp. Parasitol. 30:11–21. 10.1016/0014-4894(71)90064-6 5157122

[bib19] Harris N.L., and Loke P. 2017 Recent Advances in Type-2-Cell-Mediated Immunity: Insights from Helminth Infection. Immunity. 47:1024–1036. 10.1016/j.immuni.2017.11.015 29262347

[bib20] Hasnain S.Z., Wang H., Ghia J.E., Haq N., Deng Y., Velcich A., Grencis R.K., Thornton D.J., and Khan W.I. 2010 Mucin gene deficiency in mice impairs host resistance to an enteric parasitic infection. Gastroenterology. 138:1763–1771. 10.1053/j.gastro.2010.01.045 20138044 PMC3466424

[bib21] Hasnain S.Z., Evans C.M., Roy M., Gallagher A.L., Kindrachuk K.N., Barron L., Dickey B.F., Wilson M.S., Wynn T.A., Grencis R.K., and Thornton D.J. 2011 Muc5ac: a critical component mediating the rejection of enteric nematodes. J. Exp. Med. 208:893–900. 10.1084/jem.20102057 21502330 PMC3092342

[bib22] Howitt M.R., Lavoie S., Michaud M., Blum A.M., Tran S.V., Weinstock J.V., Gallini C.A., Redding K., Margolskee R.F., Osborne L.C., 2016 Tuft cells, taste-chemosensory cells, orchestrate parasite type 2 immunity in the gut. Science. 351:1329–1333. 10.1126/science.aaf1648 26847546 PMC5528851

[bib23] Huang Y., Guo L., Qiu J., Chen X., Hu-Li J., Siebenlist U., Williamson P.R., Urban J.F. Jr., and Paul W.E. 2015 IL-25-responsive, lineage-negative KLRG1(hi) cells are multipotential ‘inflammatory’ type 2 innate lymphoid cells. Nat. Immunol. 16:161–169. 10.1038/ni.3078 25531830 PMC4297567

[bib24] Huang Y., Mao K., Chen X., Sun M.A., Kawabe T., Li W., Usher N., Zhu J., Urban J.F. Jr., Paul W.E., and Germain R.N. 2018 S1P-dependent interorgan trafficking of group 2 innate lymphoid cells supports host defense. Science. 359:114–119. 10.1126/science.aam5809 29302015 PMC6956613

[bib25] Johansson M.E.V., Sjövall H., and Hansson G.C. 2013 The gastrointestinal mucus system in health and disease. Nat. Rev. Gastroenterol. Hepatol. 10:352–361. 10.1038/nrgastro.2013.35 23478383 PMC3758667

[bib26] Kennedy M.W. 1980 Immunologically mediated, non-specific interactions between the intestinal phases of Trichinella spiralis and Nippostrongylus brasiliensis in the mouse. Parasitology. 80:61–72. 10.1017/S0031182000000512 7383710

[bib27] Khan W.I., Blennerhasset P., Ma C., Matthaei K.I., and Collins S.M. 2001 Stat6 dependent goblet cell hyperplasia during intestinal nematode infection. Parasite Immunol. 23:39–42. 10.1046/j.1365-3024.2001.00353.x 11136476

[bib28] Klose C.S.N., and Artis D. 2016 Innate lymphoid cells as regulators of immunity, inflammation and tissue homeostasis. Nat. Immunol. 17:765–774. 10.1038/ni.3489 27328006

[bib29] Kuperman D.A., Huang X., Koth L.L., Chang G.H., Dolganov G.M., Zhu Z., Elias J.A., Sheppard D., and Erle D.J. 2002 Direct effects of interleukin-13 on epithelial cells cause airway hyperreactivity and mucus overproduction in asthma. Nat. Med. 8:885–889. 10.1038/nm734 12091879

[bib30] Maizels R.M., and McSorley H.J. 2016 Regulation of the host immune system by helminth parasites. J. Allergy Clin. Immunol. 138:666–675. 10.1016/j.jaci.2016.07.007 27476889 PMC5010150

[bib31] Maizels R.M., Hewitson J.P., Murray J., Harcus Y.M., Dayer B., Filbey K.J., Grainger J.R., McSorley H.J., Reynolds L.A., and Smith K.A. 2012 Immune modulation and modulators in Heligmosomoides polygyrus infection. Exp. Parasitol. 132:76–89. 10.1016/j.exppara.2011.08.011 21875581 PMC6485391

[bib32] McDermott J.R., Humphreys N.E., Forman S.P., Donaldson D.D., and Grencis R.K. 2005 Intraepithelial NK cell-derived IL-13 induces intestinal pathology associated with nematode infection. J. Immunol. 175:3207–3213. 10.4049/jimmunol.175.5.3207 16116211

[bib33] McKenzie A.N., Culpepper J.A., de Waal Malefyt R., Brière F., Punnonen J., Aversa G., Sato A., Dang W., Cocks B.G., Menon S., 1993 Interleukin 13, a T-cell-derived cytokine that regulates human monocyte and B-cell function. Proc. Natl. Acad. Sci. USA. 90:3735–3739. 10.1073/pnas.90.8.3735 8097324 PMC46376

[bib34] Meyer-Hoffert U., Hornef M.W., Henriques-Normark B., Axelsson L.G., Midtvedt T., Pütsep K., and Andersson M. 2008 Secreted enteric antimicrobial activity localises to the mucus surface layer. Gut. 57:764–771. 10.1136/gut.2007.141481 18250125

[bib35] Miller H.R. 1987 Gastrointestinal mucus, a medium for survival and for elimination of parasitic nematodes and protozoa. Parasitology. 94(S1, Suppl):S77–S100. 10.1017/S0031182000085838 3295692

[bib36] Monticelli L.A., Sonnenberg G.F., Abt M.C., Alenghat T., Ziegler C.G., Doering T.A., Angelosanto J.M., Laidlaw B.J., Yang C.Y., Sathaliyawala T., 2011 Innate lymphoid cells promote lung-tissue homeostasis after infection with influenza virus. Nat. Immunol. 12:1045–1054. 10.1038/ni.2131 21946417 PMC3320042

[bib37] Moro K., Yamada T., Tanabe M., Takeuchi T., Ikawa T., Kawamoto H., Furusawa J., Ohtani M., Fujii H., and Koyasu S. 2010 Innate production of T(H)2 cytokines by adipose tissue-associated c-Kit(+)Sca-1(+) lymphoid cells. Nature. 463:540–544. 10.1038/nature08636 20023630

[bib38] Neill D.R., Wong S.H., Bellosi A., Flynn R.J., Daly M., Langford T.K., Bucks C., Kane C.M., Fallon P.G., Pannell R., 2010 Nuocytes represent a new innate effector leukocyte that mediates type-2 immunity. Nature. 464:1367–1370. 10.1038/nature08900 20200518 PMC2862165

[bib39] Oeser K., Schwartz C., and Voehringer D. 2015 Conditional IL-4/IL-13-deficient mice reveal a critical role of innate immune cells for protective immunity against gastrointestinal helminths. Mucosal Immunol. 8:672–682. 10.1038/mi.2014.101 25336167

[bib40] Pichery M., Mirey E., Mercier P., Lefrancais E., Dujardin A., Ortega N., and Girard J.-P. 2012 Endogenous IL-33 is highly expressed in mouse epithelial barrier tissues, lymphoid organs, brain, embryos, and inflamed tissues: in situ analysis using a novel Il-33-LacZ gene trap reporter strain. J. Immunol. 188:3488–3495. 10.4049/jimmunol.1101977 22371395

[bib41] Portal C., Gouyer V., Magnien M., Plet S., Gottrand F., and Desseyn J.L. 2017 In vivo imaging of the Muc5b gel-forming mucin. Sci. Rep. 7:44591 10.1038/srep44591 28294161 PMC5353722

[bib42] Price A.E., Liang H.E., Sullivan B.M., Reinhardt R.L., Eisley C.J., Erle D.J., and Locksley R.M. 2010 Systemically dispersed innate IL-13-expressing cells in type 2 immunity. Proc. Natl. Acad. Sci. USA. 107:11489–11494. 10.1073/pnas.1003988107 20534524 PMC2895098

[bib43] Roy M.G., Livraghi-Butrico A., Fletcher A.A., McElwee M.M., Evans S.E., Boerner R.M., Alexander S.N., Bellinghausen L.K., Song A.S., Petrova Y.M., 2014 Muc5b is required for airway defence. Nature. 505:412–416. 10.1038/nature12807 24317696 PMC4001806

[bib44] Spits H., Artis D., Colonna M., Diefenbach A., Di Santo J.P., Eberl G., Koyasu S., Locksley R.M., McKenzie A.N.J., Mebius R.E., 2013 Innate lymphoid cells--a proposal for uniform nomenclature. Nat. Rev. Immunol. 13:145–149. 10.1038/nri3365 23348417

[bib45] Thim L., Madsen F., and Poulsen S.S. 2002 Effect of trefoil factors on the viscoelastic properties of mucus gels. Eur. J. Clin. Invest. 32:519–527. 10.1046/j.1365-2362.2002.01014.x 12153553

[bib46] Thornton D.J., Rousseau K., and McGuckin M.A. 2008 Structure and function of the polymeric mucins in airways mucus. Annu. Rev. Physiol. 70:459–486. 10.1146/annurev.physiol.70.113006.100702 17850213

[bib47] Urban J.F. Jr., Noben-Trauth N., Donaldson D.D., Madden K.B., Morris S.C., Collins M., and Finkelman F.D. 1998 IL-13, IL-4Ralpha, and Stat6 are required for the expulsion of the gastrointestinal nematode parasite Nippostrongylus brasiliensis. Immunity. 8:255–264. 10.1016/S1074-7613(00)80477-X 9492006

[bib48] Vaishnava S., Yamamoto M., Severson K.M., Ruhn K.A., Yu X., Koren O., Ley R., Wakeland E.K., and Hooper L.V. 2011 The antibacterial lectin RegIIIgamma promotes the spatial segregation of microbiota and host in the intestine. Science. 334:255–258. 10.1126/science.1209791 21998396 PMC3321924

[bib49] von Moltke J., Ji M., Liang H.-E., and Locksley R.M. 2016 Tuft-cell-derived IL-25 regulates an intestinal ILC2-epithelial response circuit. Nature. 529:221–225. 10.1038/nature16161 26675736 PMC4830391

[bib50] Vreugdenhil A.C.E., Snoek A.M.P., Greve J.W.M., and Buurman W.A. 2000 Lipopolysaccharide-binding protein is vectorially secreted and transported by cultured intestinal epithelial cells and is present in the intestinal mucus of mice. J. Immunol. 165:4561–4566. 10.4049/jimmunol.165.8.4561 11035097

[bib51] Wakelin D., and Lloyd M. 1976 Accelerated expulsion of adult Trichinella spiralis in mice given lymphoid cells and serum from infected donors. Parasitology. 72:307–315. 10.1017/S0031182000049507 967521

[bib52] Walker J.A., Barlow J.L., and McKenzie A.N.J. 2013 Innate lymphoid cells-how did we miss them? Nat. Rev. Immunol. 13:75–87. 10.1038/nri3349 23292121

[bib53] Wang C.H., and Bell R.G. 1986 Trichinella spiralis: newborn larval migration route in rats reexamined. Exp. Parasitol. 61:76–85. 10.1016/0014-4894(86)90137-2 3943594

[bib54] Webb R.A., Hoque T., and Dimas S. 2007 Expulsion of the gastrointestinal cestode, Hymenolepis diminuta by tolerant rats: evidence for mediation by a Th2 type immune enhanced goblet cell hyperplasia, increased mucin production and secretion. Parasite Immunol. 29:11–21. 10.1111/j.1365-3024.2006.00908.x 17187651

[bib55] Wells J.M., Brummer R.J., Derrien M., MacDonald T.T., Troost F., Cani P.D., Theodorou V., Dekker J., Méheust A., de Vos W.M., 2017 Homeostasis of the gut barrier and potential biomarkers. Am. J. Physiol. Gastrointest. Liver Physiol. 312:G171–G193. 10.1152/ajpgi.00048.2015 27908847 PMC5440615

[bib56] Young H.W.J., Williams O.W., Chandra D., Bellinghausen L.K., Pérez G., Suárez A., Tuvim M.J., Roy M.G., Alexander S.N., Moghaddam S.J., 2007 Central role of Muc5ac expression in mucous metaplasia and its regulation by conserved 5′ elements. Am. J. Respir. Cell Mol. Biol. 37:273–290. 10.1165/rcmb.2005-0460OC 17463395 PMC1994232

[bib57] Zaph C., Cooper P.J., and Harris N.L. 2014 Mucosal immune responses following intestinal nematode infection. Parasite Immunol. 36:439–452. 10.1111/pim.12090 25201407 PMC4312905

