# Supplementary material for: ILC2s mediate systemic innate protection by priming mucus production at distal mucosal sites
Source: J Exp Med. 2019 Oct 3;216(12):2714–23. doi: 10.1084/jem.20180610 (PMC6888984; doi:10.1084/jem.20180610)
Supplement: Supplemental Materials (PDF) [file JEM_20180610_sm.pdf]

Supplemental material

Campbell et al., https://doi.org/10.1084/jem.20180610

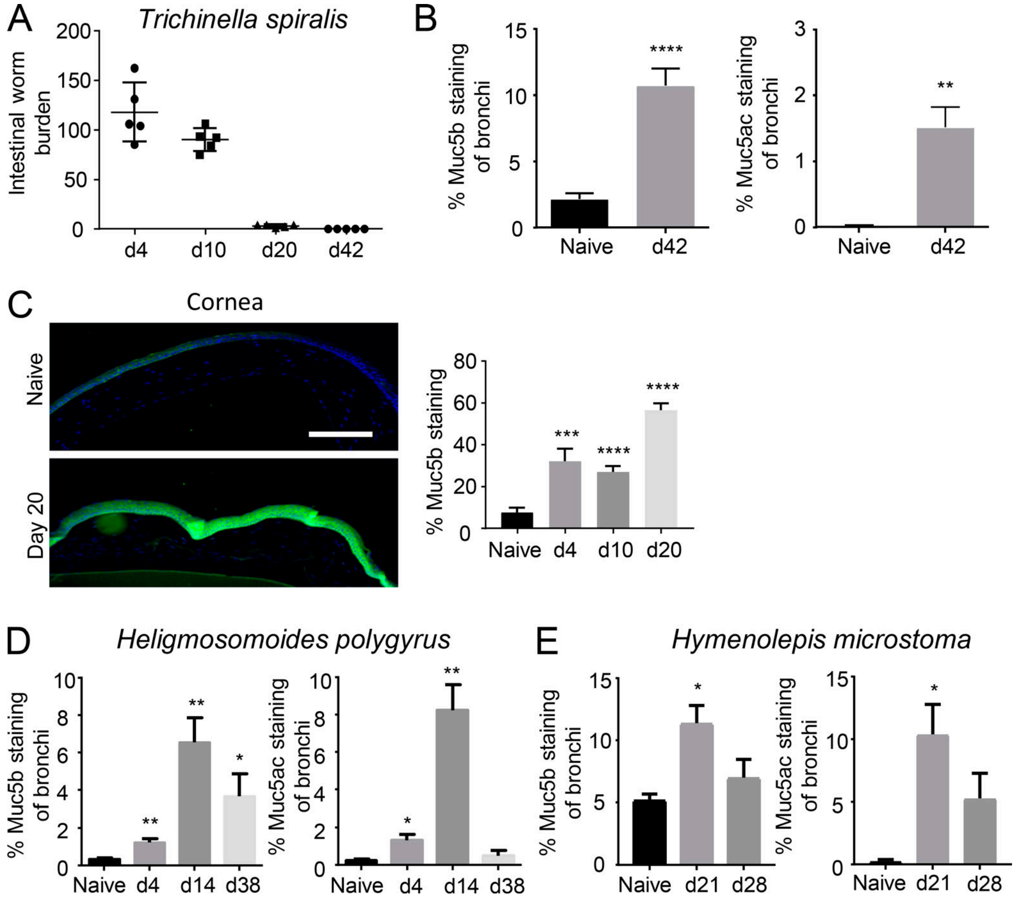

Figure S1. ***T. spiralis*** **expulsion kinetics and systemic mucus responses across peripheral mucosal sites after intestinal dwelling helminth infection.** **(A)** *T. spiralis* intestinal worm burden p.i. **(B)** Quantification of lung sections stained for Muc5b and Muc5ac from naive and day 42 *T. spiralis*–infected C57/BL6 mice (*n* = 5 mice/group). **(C)** Representative cornea sections from naive and day 20 *T. spiralis*–infected C57/BL6 mice stained for Muc5b (bar, 100 µm) and corresponding quantification of staining (*n* = 5 mice/group). **(D)** C57/BL6 mice were infected with *H. polygyrus*, and lung sections were stained and quantified for Muc5b and Muc5ac at days 4, 14, and 38 p.i. (*n* = 5 mice/group). **(E)** C57/BL6 mice were infected with *H. microstoma*, and lung sections were stained and quantified for Muc5b and Muc5ac at days 21 and 28 p.i. (*n* = 3 mice/group). Data are representative of two independent experiments. Error bars indicate the mean ± SEM. Comparisons between groups were calculated using either unpaired Student's *t* tests (A) or a one-way ANOVA and Sidak's post-test (B–D). *, P ≤ 0.05; **, P ≤ 0.01; ***, P ≤ 0.001; ****, P ≤ 0.0001.

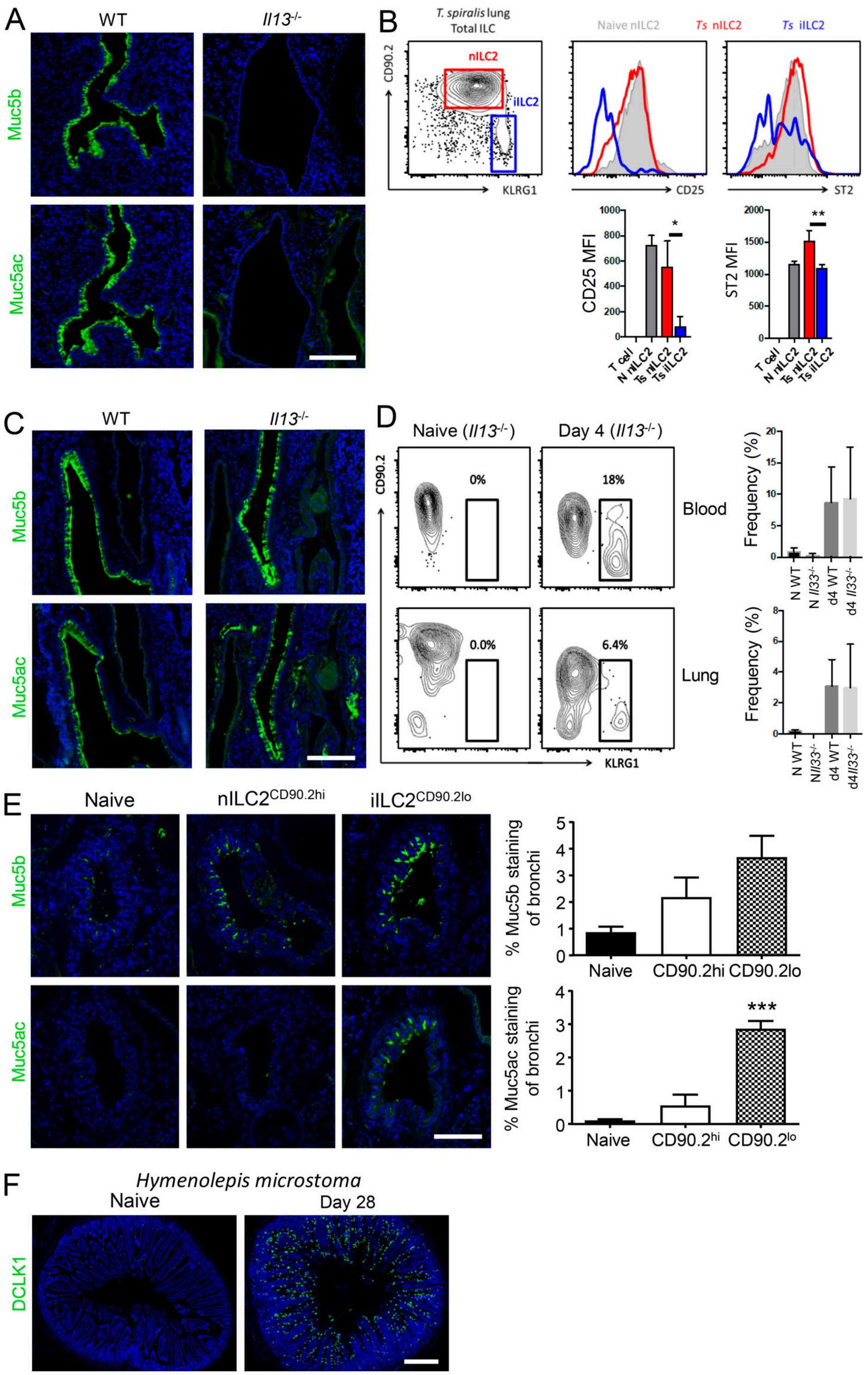

Figure S2. **IL-13, but not IL-33, is essential for driving the lung mucus response during *T. spiralis* infection. (A)** Representative lung sections from day 10–infected WT and IL-$13^{eGFP/eGFP}$ mice stained for either Muc5b (top panel) or Muc5ac (bottom panel); bar, 200 µm. **(B)** Lung cells from naive and day 4–infected C57BL6 mice were analyzed by flow cytometry for ILC2 markers (nILC2 gated as Lin^- $CD127^+$ $CD90.2^{hi}$ $KLRG1^{int}$; iILC2 gated as Lin^- $CD127^+$ $CD90.2^{lo}$ $KLRG1^{hi}$) and expression of CD25 and ST2 analyzed for each ILC2 population. Ts, *T. spiralis*; MFI, mean fluorescence intensity. **(C)** Representative lung sections from day 10–infected WT and *IL-33*$^{-/-}$ mice stained for either Muc5b (top panel) or Muc5ac (bottom panel); bar, 200 µm. **(D)** Cells from the blood and lung of naive and day 4–infected WT and *IL-33*$^{-/-}$ mice were analyzed by flow cytometry for iILC2 markers (gated as Lin^- $CD127^+$ $CD90.2^{lo}$ $KLRG1^{hi}$). **(E)** iILC2 are sufficient to transfer elevated mucin responses to the lungs of NOD/SCIDγ$c^{-/-}$ mice; representative lung sections stained for Muc5b or Muc5ac and corresponding quantification. Mucin response in bronchi following transfer of 5 × 10^4 IL-13^{eGFP+} $nILC2^{CD90.2hi}$ or $iILC2^{CD90.2lo}$ taken from lungs of IL13eGFP reporter mice infected with *T. spiralis* on day 10 p.i. Data taken on day 8 after cell transfer (bars, 200 µm). **(F)** Representative jejunum sections from *H. microstoma*–infected mice stained for the tuft cell marker DCLK1 (bar, 400 µm). Data are representative of two independent experiments. Error bars indicate the mean ± SEM. Comparisons between groups were calculated using one-way ANOVA and Sidak's post-test (B). *, $P \le 0.05$; **, $P \le 0.01$; ***, $P \le 0.001$.

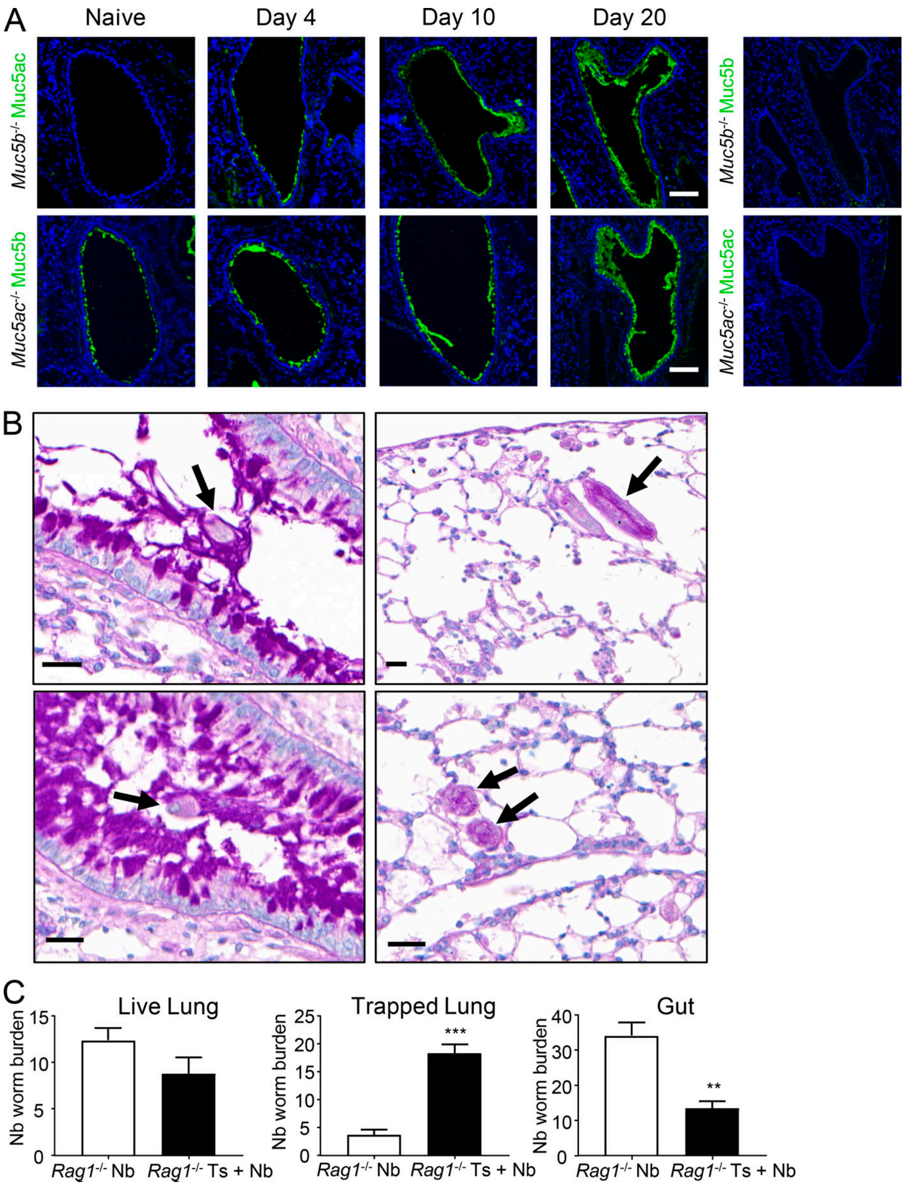

Figure S3. **Mucin compensation, lung trapping of Nb, and protective immunity in immunodeficient mice. (A)** Representative lung sections from *T. spiralis*–infected *Muc5b*$^{-/-}$ or *Muc5ac*$^{-/-}$ mice stained for either Muc5ac (top panel) or Muc5b (bottom panel), with corresponding control sections (bars, 100 µm). **(B)** Representative PAS staining of lung tissue sections from coinfected C57BL6 mice (*T. spiralis* and Nb) showing Nb larvae (arrowed) within mucus (left panels) and within lung parenchyma (right panels; bar, 20 µm). **(C)** Naive (white bars) or day 20 after *T. spiralis* (Ts) infection (black bars) *Rag1*$^{-/-}$ mice were i.v. infected with Nb. Total Nb worm numbers were collected at day 3 after Nb infection from lung tissue (left), collagenase-digested lung tissue (middle), and small intestine (right; n = 4 mice/group). Data are representative of two (A and C) or four (B) independent experiments. Error bars indicate the mean ± SEM. Comparisons between groups were calculated using unpaired Student's t tests. **, $P \leq 0.01$; ***, $P \leq 0.001$.

https://doi.org/10.1084/jem.20180610
